# Predictors of Recurrence after Catheter Ablation of Paroxysmal Atrial Fibrillation in Different Follow-Up Periods

**DOI:** 10.3390/medicina56090465

**Published:** 2020-09-11

**Authors:** Masako Baba, Kentaro Yoshida, Yoshihisa Naruse, Ai Hattori, Yoshiaki Yui, Akira Kimata, Yoko Ito, Yasuaki Tsumagari, Hidekazu Tsuneoka, Yasutoshi Shinoda, Tomohiko Harunari, Yuichi Hanaki, Hideyuki Hasebe, Masako Misaki, Daisuke Abe, Akihiko Nogami, Masaki Ieda, Noriyuki Takeyasu

**Affiliations:** 1 Department of Cardiology, Ibaraki Prefectural Central Hospital, Kasama 309-1793, Japan; babamasako1010@yahoo.co.jp (M.B.); naru_4413@yahoo.co.jp (Y.N.); aihattori0141@gmail.com (A.H.); yyui541026@yahoo.co.jp (Y.Y.); a_kimata_414@yahoo.co.jp (A.K.); happa_y0714@yahoo.co.jp (Y.I.); yaaasuaki520@yahoo.co.jp (Y.T.); yuhurate0123@yahoo.co.jp (H.T.); shinocchi1029@yahoo.co.jp (Y.S.); t_harunari2000@yahoo.co.jp (T.H.); yu_hana1232@hotmail.com (Y.H.); EMUEMU0006@aol.com (M.M.); daisuke-a@mtg.biglobe.ne.jp (D.A.); n-takeyasu@chubyoin.pref.ibaraki.jp (N.T.); 2 Department of Cardiology, Faculty of Medicine, University of Tsukuba, Tsukuba 305-8575, Japan; h153478@siz.saiseikai.or.jp (H.H.); anogami@md.tsukuba.ac.jp (A.N.); mieda@md.tsukuba.ac.jp (M.I.)

**Keywords:** atrial fibrillation, ablation, recurrence, natriuretic peptide, remodeling

## Abstract

*Background and objectives:* Pulmonary vein (PV) reconnection is a major reason for recurrence after catheter ablation of paroxysmal atrial fibrillation (PAF). However, the timing of the recurrence varies between patients, and recurrence >1 year after ablation is not uncommon. We sought to elucidate the characteristics of atrial fibrillation (AF) that recurred in different follow-up periods. *Materials and Methods:* Study subjects comprised 151 consecutive patients undergoing initial catheter ablation of PAF. Left atrial volume index (LAVi) and atrial/brain natriuretic peptide (ANP/BNP) levels were systematically measured annually over 3 years until AF recurred. *Results:* Study subjects were classified into four groups: non-recurrence group (*n* = 84), and short-term- (within 1 year) (*n* = 30), mid-term- (1–3 years) (*n* = 26), and long-term-recurrence group (>3 years) (*n* = 11). The short-term-recurrence group was characterized by a higher prevalence of diabetes mellitus (hazard ratio 2.639 (95% confidence interval, 1.174–5.932), *p* = 0.019 by the Cox method), frequent AF episodes (≥1/week) before ablation (4.038 (1.545–10.557), *p* = 0.004), and higher BNP level at baseline (per 10 pg/mL) (1.054 (1.029–1.081), *p* < 0.0001). The mid-term-recurrence group was associated with higher BNP level (1.163 (1.070–1.265), *p* = 0.0004), larger LAVi (mL/m^2^) (1.033 (1.007–1.060), *p* = 0.013), and longer AF cycle length at baseline (per 10 ms) (1.194 (1.058–1.348), *p* = 0.004). In the long-term-recurrence group, the ANP and BNP levels were low throughout follow-up, as with those in the non-recurrence group, and AF cycle length was shorter (0.694 (0.522–0.924), *p* = 0.012) than those in the other recurrence groups. *Conclusions:* Distinct characteristics of AF were found according to the time to first recurrence after PAF ablation. The presence of secondary factors beyond PV reconnections could be considered as mechanisms for the recurrence of PAF in each follow-up period.

## 1. Introduction

Catheter ablation has become an established treatment for atrial fibrillation (AF), and pulmonary vein (PV) isolation is a cornerstone of procedural strategy targeting AF. However, the success rate after the first ablation procedure is still modest (~70% during short-term follow-up), and approximately 50% of patients require multiple procedures during long-term follow-up [1]. As many previous studies have confirmed, PV reconnections are a major reason for the recurrence of paroxysmal AF during short-term follow-up [1,2] and can potentially occur in an early period because acute edema occurring after radiofrequency lesions are created is resolved within 2 weeks, and lesion maturation is completed within the blanking period of 3 months [3,4]. However, the timing of the recurrence of AF varies between patients, and recurrence >1 year after ablation is not uncommon [5,6,7]. This suggests that mechanisms beyond PV reconnections are associated with the timing of the recurrence of AF [5,6]. In the present study, we sought to investigate differences in the characteristics of recurrence between short-term (~1 year), mid-term (1 to 3 years), and long-term (>3 years) follow-up periods after catheter ablation of paroxysmal AF. To do so, echocardiographic variables and atrial/brain natriuretic peptide (ANP/BNP) levels were systematically measured over 3 years [8].

## 2. Materials and Methods

### 2.1. Study Subjects

This study included 151 consecutive patients referred to Ibaraki Prefectural Central Hospital for initial catheter ablation of paroxysmal AF from April 2012 to February 2017. Patients with an estimated glomerular filtration rate <30 mL/min, patients with moderate to severe valvular diseases, and patients with symptomatic heart failure (New York Heart Association Classification ≥III) were excluded from the study. The current study was conducted in accordance with the Declaration of Helsinki and was approved by the Institutional Review Board and Ethics Committee of Ibaraki Prefectural Central Hospital, Japan (No. 453) on 16 May 2017.

### 2.2. Cardiac Biomarkers

Levels of plasma ANP and BNP were measured within 1 month before catheter ablation. Plasma ANP level was measured with a chemiluminescent enzyme immunoassay (CLEIA), and the BNP level was also measured with a chemiluminescent immunoassay (CLIA) (BML, Inc., Tokyo, Japan) [8,9]. After ablation, these biomarkers were systematically measured annually until recurrence of AF was documented and a redo procedure was performed.

### 2.3. Echocardiographic Evaluations

Left atrial volume (indexed) (LAVi), left ventricular ejection fraction, and E/e’ were evaluated before ablation, between 6 months and 12 months after ablation, and annually thereafter until recurrence of AF was documented. The LA volumes were measured on apical two- and four-chamber views during end-systole via the biplane method [10].

### 2.4. Measurement of AF Cycle Length in Lead V1

**Figure 1:**
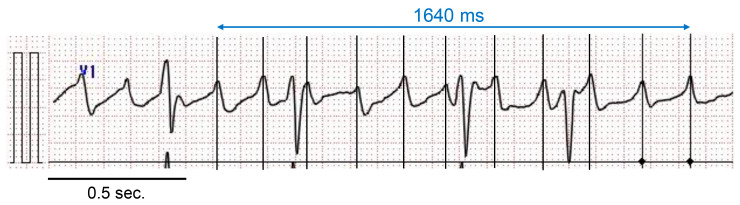
Measurement of the atrial fibrillation cycle length in lead V1 (AFCL_V1_). The electrocardiogram (ECG) was magnified at 50 mm/s and 20 mm/mV. The AFCL_V1_ calculated in this patient was 164 ms.

AF cycle length in the surface electrocardiogram (ECG) lead V1 was manually measured from 10 unambiguous fibrillatory waves using electric calipers (Physiological Examination System, PRM4241 Ver. 05-03; Nihon Kohden, Tokyo, Japan) by a physician blinded to the patient’s history (M.B.) (Figure 1) [11,12]. The mean value calculated from the 10 cycle lengths was defined as AFCL_V1_. Twenty patients were selected at random for the assessments of the interobserver reproducibility of the AFCL_V1_ measurements by M.B. and K.Y.

### 2.5. Catheter Ablation

An ECG-gated 256-slice computed tomography scan of the heart was performed within 7 days before the ablation procedure (SOMATOM Definition Flash, Siemens, Erlangen, Germany). The left atrium (LA) and PVs were reconstructed with three-dimensional segmentation software using a CARTO 3 system (Biosense Webster, Diamond Bar, CA, USA). Electrophysiological study and catheter ablation proceeded under conscious sedation with dexmedetomidine and fentanyl. Surface ECGs and intracardiac electrograms were continuously monitored and stored on an EP-WorkMate recording system (Abbott, Saint Paul, MN, USA). A 6F 20-pole dual-site mapping catheter (BeeAT; Japan Lifeline Co., Ltd., Tokyo, Japan) was inserted through the subclavian vein and positioned in the coronary sinus (CS), right atrium, and superior vena cava (SVC) throughout the procedure. An intracardiac echocardiography catheter (AcuNav, Biosense Webster) was advanced to the right atrium via the femoral approach to guide the transseptal puncture. Two long sheaths (SL0; AF Division, Abbott) were then advanced into the LA.

Antral PV isolation was performed in all patients with guidance from a three-dimensional mapping system (CARTO, Biosense-Webster or EnSite NavX, Abbott). A bolus of unfractionated heparin was administered before the first transseptal puncture, and the infusion was titrated to maintain an activated clotting time between 300–350 s. Circumferential ablation was applied within the antrum of the LA. Radiofrequency current was delivered via a ThermoCool (Biosense-Webster) or Cool Path Duo (Abbott) catheter with power up to 30 W. The endpoint was the achievement of a bidirectional conduction block between the LA and the PVs. Thereafter, a cavotricuspid isthmus line was created, with an endpoint of bidirectional conduction block [7]. If non-PV ectopies were reproducibly observed with and without the continuous infusion of isoproterenol (1–4 mcg/min), they were targeted for ablation [13,14,15,16,17]. Neither ablation of continuous fractionated atrial electrograms nor left atrial linear ablation was performed in this series in the index procedures.

### 2.6. Follow-Up

Patients were discharged on anticoagulation at ≥3 days after ablation. All antiarrhythmic drugs administered before the procedure were discontinued in all patients. However, the use of antiarrhythmic drugs was allowed only during the blanking period of 3 months according to the physician’s discretion. The first outpatient clinic visit with 12-lead ECG recording was scheduled around 2 weeks after the procedure and then every 2 months thereafter. Recordings with a 24-h Holter event recorder (DSC-3300; Nihon Kohden), an HCG-901 (Omron, Kyoto, Japan) for >30 days, or a SpiderFlash-t AFIB (SORIN, Clamart, France) auto-trigger external loop recorder for 14 to 30 days were undertaken 6 months after ablation and every 6 months thereafter. Patients were also asked to call a nurse if they experienced symptoms suggestive of an arrhythmia. In such cases, an event recorder or external loop recorder was used in addition to the routine check-ups. Transient occurrences of AF only during the blanking period were not considered as recurrence of AF, and treatment success was defined as freedom from all atrial tachyarrhythmias lasting for >30 s in the absence of antiarrhythmic drug therapy after the blanking period. Study subjects were classified into four groups: non-recurrence group, short-term recurrence group (from 3 months to 1 year), mid-term recurrence group (from 1 year to 3 years), and long-term recurrence group (over 3 years). A repeat procedure was recommended in patients with recurrent atrial tachyarrhythmia.

### 2.7. Statistical Analysis

Continuous variables are expressed as mean ± standard deviation (SD) or median [25th-75th percentile] and were compared by Student *t*-test or Mann–Whitney U-test, as appropriate. Categorical variables are reported as number and percentage and were compared by Chi-square analysis or with Fisher’s exact test, as appropriate. One-way analysis of variance (ANOVA) or the Kruskal–Wallis test was used to compare the continuous results from the four groups. When significant differences between groups were present, the Scheffé test or Steel–Dwass test was used to compare individual groups. The serial change in the LAVi during the follow-up was assessed by repeated measures of ANOVA. Univariate and multivariate analyses of variables for arrhythmia recurrence were performed by the Cox method for the three individually determined hazard phases of short-, mid-, and long-term recurrence. A two-tailed *p* value of <0.05 indicated statistical significance. All statistical analyses were carried out using JMP version 12.0 (SAS Institute Inc., Cary, NC, USA).

## 3. Results

### 3.1. Patient Characteristics

All PVs were successfully isolated. After PV isolation, continuous infusion of isoproterenol was performed to induce non-PV ectopies, and those were targeted for ablation (SVC isolation in 13 patients and ablation of the atrial septum in 4, coronary sinus in 7, and patent left SVC in 2 patients).

**Figure 2:**
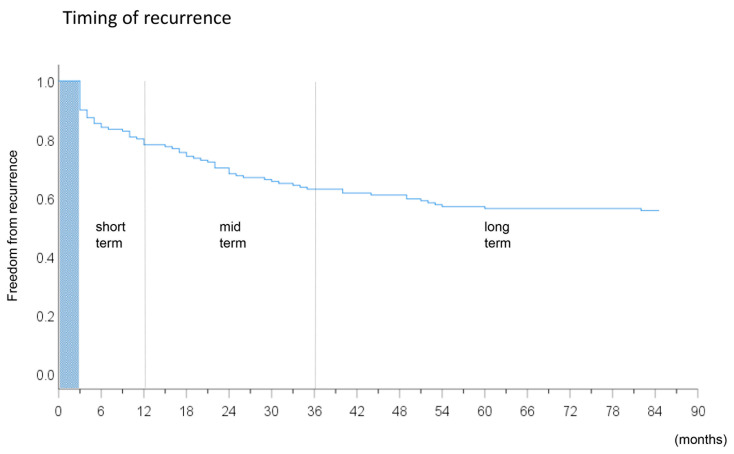
Kaplan–Meier curve revealing the timing of recurrences of atrial fibrillation in all study subjects after the index procedure.

The numbers of patients in the non-recurrence group, short-term recurrence group, mid-term recurrence group, and long-term recurrence group were 84 (56%), 30 (20%), 26 (17%), and 11 (7%), respectively. The patient characteristics and Kaplan–Meier curve of the study subjects are presented in Table 1 and Figure 2, respectively. The prevalence of overall non-PV triggers was comparable between the four patient groups (12 (14%), 5 (17%), 6 (23%), and 3 (27%), respectively), *p* = 0.77), and the prevalence in each location of the non-PV triggers was also comparable between the groups (*p* > 0.05 for all).

**Figure 3:**
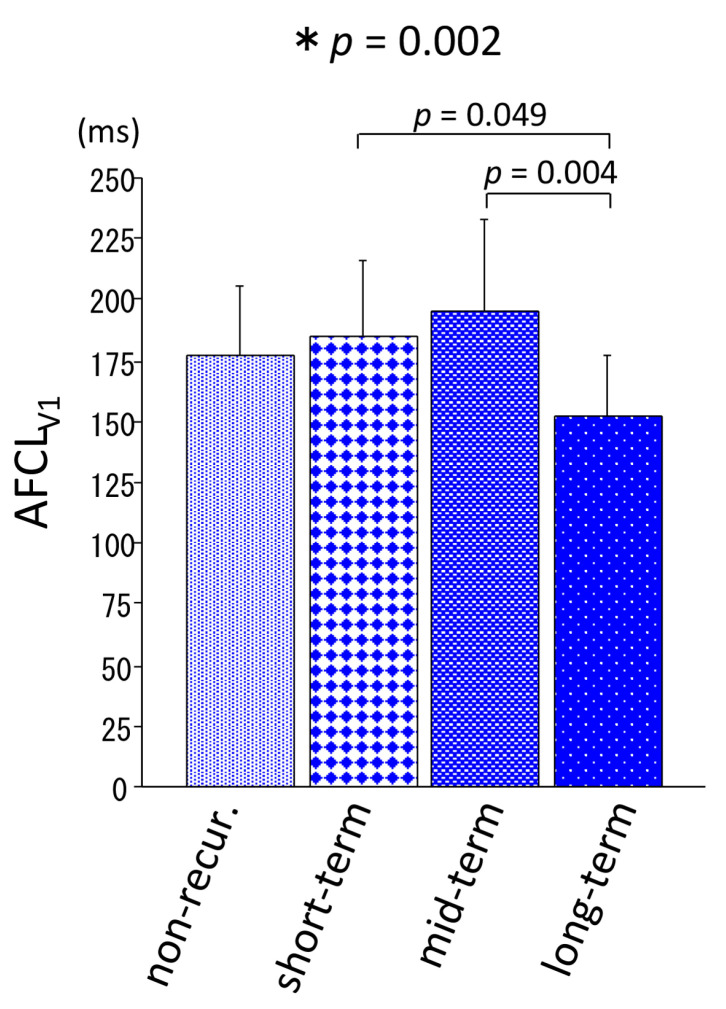
Atrial fibrillation cycle length in lead V1 (AFCL_V1_) measurement before ablation in the non-recurrence and short-term, mid-term, and long-term recurrence groups. The bars indicate mean and SD. * ANOVA. *AFCL_V1_* atrial fibrillation cycle length in lead V1.

Diabetes mellitus was defined as an HbA1c of ≥6.5% and/or taking hypoglycemic agents and was more prevalent in the short-term recurrence group than in the other groups. The details of medications before ablation are also provided in Table 1. Frequent AF episodes before ablation (≥1 episode per week) were more common in the short-term recurrence group (*p* = 0.005). Interobserver variability had an acceptable degree of reproducibility, as reflected by a correlation coefficient of 0.94 for the AFCL_V1_ measurements between the two investigators. The AFCL_V1_ was significantly shorter in the long-term recurrence group than that in the other three groups (177 ± 29 vs. 184 ± 31 vs. 195 ± 37 vs. 152 ± 24 ms, *p* = 0.002) (Figure 3).

### 3.2. Serial Measurements of ANP/BNP Levels

**Figure 4:**
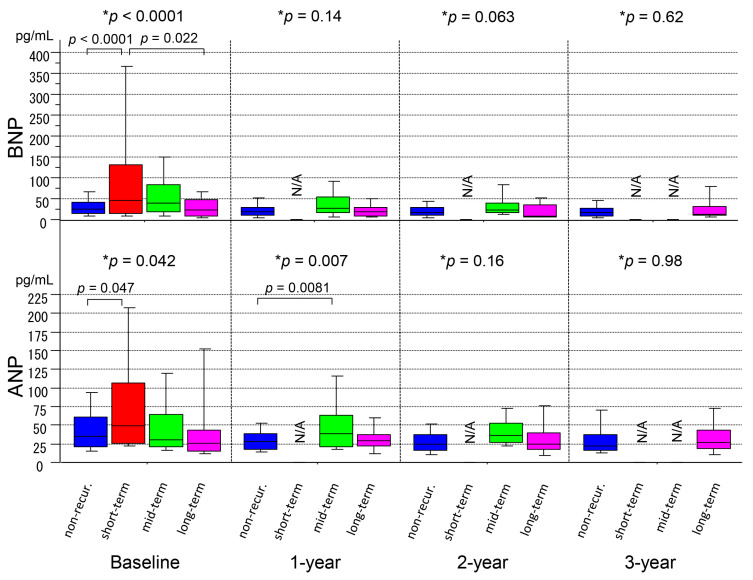
Serial measurements of the brain natriuretic peptide (BNP) and atrial natriuretic peptide (ANP) levels for 3 years after ablation in the non-recurrence and short-term, mid-term, and long-term recurrence groups. The boxes indicate median and interquartile range, and bars indicate minimum and maximum values. * Kruskal–Wallis Test. *ANP* atrial natriuretic peptide, *BNP* brain natriuretic peptide.

The BNP level at baseline was significantly higher in the short-term recurrence group than that in the non-recurrence group or long-term recurrence group (*p* < 0.0001), whereas those at the 1-year, 2-year, and 3-year follow-ups did not show significant differences between the groups (*p* = 0.14, *p* = 0.063, and *p* = 0.62, respectively) (Figure 4 and Table 2). It was noted that the BNP level in the long-term recurrence group was low throughout the follow-up period, as was that in the non-recurrence group. The ANP level showed a similar trend as the BNP level. However, that at 1 year also revealed a statistically significant difference between the groups (*p* = 0.007) (Figure 4 and Table 2).

### 3.3. Serial Measurements of LAVi

**Figure 5:**
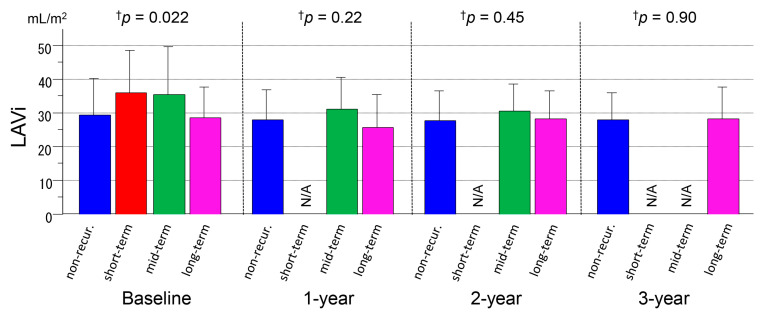
Serial measurements of the left atrial volume indexed (LAVi) for 3 years after ablation in the non-recurrence and short-term, mid-term, and long-term recurrence groups. † ANOVA. *LAVi* left atrial volume (indexed), *N/A* not available.

Although the LAVi at baseline was significantly different between the groups (*p* = 0.022, ANOVA), the comparisons of the individual groups did not show significant differences in the LAVi in the 1-year, 2-year, and 3-year follow-ups (*p* = 0.22, *p* = 0.45, and *p* = 0.90, respectively) (Figure 5 and Table 2). In this series with paroxysmal AF and a structurally normal heart, reversal of structural remodeling, i.e., reduction in the LAVi during the follow-up, was not statistically apparent (*p* = 0.21, NA, *p* = 0.70, and *p* = 0.18, respectively). The LAVi correlated with the ANP level (*R* = 0.34, *p* < 0.001) and BNP level (*R* = 0.51, *p* < 0.001).

### 3.4. Predictors of AF Recurrence at Different Time-Points

**Figure 6:**
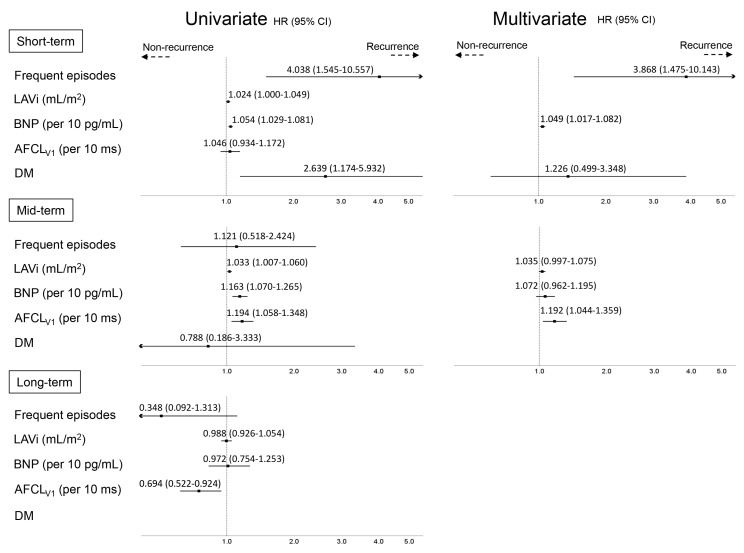
Univariate and multivariate analyses of variables for arrhythmia recurrence by the Cox method. *AFCL_V1_* atrial fibrillation cycle length in lead V1, *BNP* brain natriuretic peptide, *DM* diabetes mellitus, *LAVi* left atrial volume indexed.

Cox regression analyses were performed separately for the three follow-up phases (Figure 6). During the short-term period, diabetes mellitus, frequent AF episodes (≥1 per week), and the BNP level at baseline were the predictors of AF recurrence in the univariate analysis (hazard ratio 2.639 (95% confidence interval 1.174–5.932), *p* = 0.019; 4.038 (1.545–10.557), *p* = 0.004; and 1.054 (1.029–1.081), *p* <0.0001, respectively), and frequent AF episodes and BNP level were the independent predictors in the multivariate analysis (3.868 (1.475–10.143), *p* = 0.006 and 1.049 (1.017–1.082), *p* = 0.002, respectively). During the mid-term period, LAVi, BNP level, and AFCL_V1_ were the predictors of AF recurrence in the univariate analysis (1.033 (1.007–1.060), *p* = 0.013; 1.163 (1.070–1.265), *p* = 0.0004; and 1.194 (1.058–1.348), *p* = 0.004, respectively), and the independent predictor of AF recurrence in the multivariate analysis was longer AFCL_V1_ (1.192 (1.044–1.359), *p* = 0.0091). During the long-term period, a shorter AFCL_V1_ (0.694 (0.522–0.924), *p* = 0.012) was the only predictor of AF recurrence in the univariate analysis (Figure 6).

### 3.5. PV Reconnections and Outcomes after 2nd Ablation Procedure

**Figure 7:**
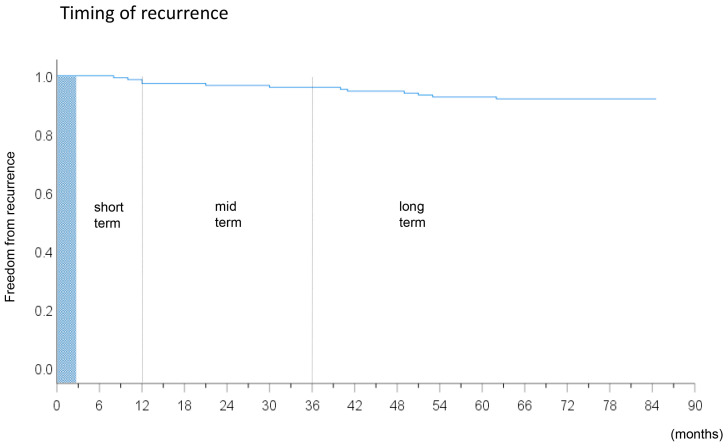
Kaplan–Meier curve revealing atrial fibrillation-free survival after one or two ablation procedures.

A second procedure was not performed in six of the 67 (9%) patients in the recurrence groups because the frequency of AF episodes had significantly decreased, and patients’ symptoms were sufficiently improved in one patient, surgical ablation was performed in one patient, and anti-tachycardia pacing with a pacemaker (Reactive ATP; Medtronic, Minneapolis, MN, USA) completely suppressed AF episodes in two patients. The reasons for the remaining two patients were non-cardiac issues such as cancer and interstitial pneumonia, respectively. The number of PV reconnections was not significantly different between the recurrence groups (56/108 (52%) PVs in 27 patients, 46/96 (48%) PVs in 24 patients, and 22/40 (55%) PVs in 10 patients, respectively, *p* = 0.81). At the second procedure, all reconnected PVs were successfully re-isolated. Non-PV targets were the SVC in 24 (40%), coronary sinus in 5 (8%), right atrial septum and appendage in 3 (5%), left atrial appendage in 3 (5%), linear ablation at the mitral isthmus in 3 (5%), and roofline ablation in 2 (3%) patients. Overall AF-free survival after one or two procedure(s) is shown in Figure 7. AF recurred after the second ablation procedure in 5 (17%) patients, 6 (23%) patients, and 1 (9%) patient, respectively, during the median follow-up period of 44 months, revealing that 139/151 (92%) patients were free from AF recurrence after a mean of 1.4 procedures.

## 4. Discussion

### 4.1. Main Findings

Time to the recurrence of AF after the first procedure was associated with electrophysiological, echocardiographic, biological, hemodynamic, and metabolic variables that can all characterize AF.

- Patients in the short-term recurrence group more frequently suffered from AF episodes and diabetes mellitus than those in the other groups and had a higher BNP level at baseline than the non-recurrence group;
- Patients in the mid-term recurrence group had a larger LA and longer AFCL_V1_ at baseline compared with the other groups;
- Patients in the long-term recurrence group were hemodynamically stable throughout the long follow-up period, as were those in the non-recurrence group, and had a shorter AFCL_V1_ at baseline;
- The overall success rate after one or two procedure(s) was satisfactory regardless of the timing of recurrence, indicating that an ablation strategy including PV isolation followed by ablation for non-PV ectopies, mainly SVC isolation, is adequately effective as a treatment of paroxysmal AF.

As is well known, PV reconnection is a major reason for the recurrence of AF. However, the presence of “second factors” beyond ectopic triggers from the PVs may be associated with the timing of recurrence after the first procedure [18].

### 4.2. Short-Term Recurrence Group

This group was characterized by impaired hemodynamic status before ablation (higher BNP level) probably due to more frequent AF episodes before ablation and an unfavorable metabolic milieu (diabetes mellitus). It is well known that the BNP level before ablation is an independent predictor for recurrence of AF during short-term follow-up (~1 year) [8,19]. Although hemodynamic status should be improved by the maintenance of sinus rhythm after ablation, electrical, structural, and autonomic remodeling associated with impaired hemodynamics (atrial and PV stretch) needs time to reverse [20]. If PV reconnection occurs and ectopic triggers appear during this early recovery phase, the atria may be more susceptible to AF than at a later phase. Furthermore, a strong relationship between diabetes mellitus, sympathetic over-activity, and AF is clear because metabolic changes lead to inflammatory reaction, endothelial dysfunction, and abnormal activation of the renin–angiotensin–aldosterone system [21,22]. Of note, the triggers of all AF episodes in the short-term recurrence group were considered to be sympathetic over-activity during the daytime. Otake et al. revealed that diabetic mice were more susceptible to AF after sympathetic stimulation than controls (autonomic remodeling) [23]. This also implies that an autonomic imbalance due to diabetes could play an essential role in the recurrence of AF early after the ablation procedure, during which sympathetic and parasympathetic activities fluctuate due to ablation of the ganglionated plexus (GP). Positive GP responses during PV isolation are also associated with the recurrence of AF [24].

### 4.3. Mid-Term Recurrence Group

This group was characterized by the dominant role of structural remodeling (larger LA) rather than electrical remodeling (longer AFCL_V1_) in the occurrence of AF. Pratola et al. performed a repeat electrophysiological study in 20 volunteers who had undergone PV isolation and had not suffered any recurrences of AF during a minimum follow-up period of 2.5 years after ablation. Of note, PV reconnection was found in 62.5% of the veins even in patients with successful long-term outcomes [2]. This implies that PV reconnection is often present but is not enough for the recurrence of AF, and second factors beyond the PVs were suggested. Substrate modification by ablation encircling the PV antra may have decreased susceptibility to AF and may delay the time of recurrence after ablation [18]. Furthermore, parasympathetic over-activity implied by intense exercise and the presence of AF episodes during nighttime may be one of the possible mechanisms for late-phase recurrences, which may be associated with the temporary effect of GP ablation. However, these factors did not reach statistical significance in the present small study.

### 4.4. Long-Term Recurrence Group

This group was characterized by little impairment to hemodynamics (lower ANP and BNP levels) as with the non-recurrence group, and advanced electrical remodeling (shorter AFCL_V1_) [25]. Although the associated mechanisms are unclear, this implied the presence of genetic variants for a short effective refractory period [12,25]. PV isolation eliminated the main triggers from the PVs that are responsible for AF, and the prevalence of AF decreased under little structural remodeling, but an incidental overlap of mental and physical stress, autonomic imbalance, and non-PV ectopies under a favorable electrical milieu may cause the very late recurrence of AF after ablation [26].

### 4.5. Prior Studies

A few studies investigated patients with recurrence of AF after long-term success (freedom from recurrence of at least 36 months). Usui et al. [5] reported 37 such patients with paroxysmal AF, in whom PV reconnections were found in 78% of the patients at a redo procedure, and AF independent of the PVs was assumed to be present in 65% of the patients, implying that both PV and non-PV arrhythmogenicities contributed to such a super-delayed recurrence. In contrast to our study, there was no comparison to patients with short- or mid-term recurrence. Shah et al. [6] also reported 137 patients with a super delayed (>36 months) recurrence. However, long-term success was achieved after multiple ablation procedures in 26% of the patients, and therefore, their study design was different from that of ours and the Usui et al. study. PV reconnection was found in 81% of their patients, and again, no direct comparison was made to patients with short- and mid-term recurrence. Both studies applied retrospective analyses and mainly focused on the procedural findings, not the characteristics of AF such as patient comorbidities, echocardiographic findings, cardiac biomarkers, and AFCL.

### 4.6. Study Limitations

First, asymptomatic events may have been missed during long-term follow-up. However, the patients were all symptomatic before ablation, and the frequent check-ups in the outpatient clinic (more than every 2 months) and the repeated use of 14- to 30-day loop recorders may have minimized the under-diagnosis of asymptomatic recurrent arrhythmias. Second, AFCL was measured only in lead V1 because more than half of the patients presented to the electrophysiological laboratory in sinus rhythm in this series with paroxysmal AF. Third, the number of patients in each group was small. The multivariate analyses in each recurrence group may have caused overfitting of the variables, and should be carefully interpreted as a preliminary result, and should be validated in a larger future study. However, to the best of our knowledge, there is no study focusing on the differences in mechanisms of recurrence of AF between different follow-up periods, and the main advantage of the present study is its longitudinal design with serial measurements of cardiac biomarkers and echocardiographic variables over 3 years.

## 5. Conclusions

Distinct predictors of AF were found according to the time to first recurrence after AF ablation. The presence of secondary factors beyond PV reconnections could be considered as mechanisms for recurrence of PAF in each follow-up period. Because this was a small preliminary study, further investigations are needed to validate the present results.

## Figures and Tables

**Table 1:** Patient characteristics.

Variables	No Recurrence (*n* = 84)	Short-Term Recurrence (*n* = 30)	Mid-Term Recurrence (*n* = 26)	Long-term Recurrence (*n* = 11)	*p* Value
Time to recurrence (mo.)		4 (3–6)	22 (17–26)	51 (45–54)	NA
Female	17 (20.2%)	6 (20.0%)	6 (23.1%)	2 (18.0%)	0.99
Age (years)	64 ± 8	63 ± 9	66 ± 7	61 ± 12	0.44
Age at onset (years)	60 ± 11	57 ± 11	62 ± 8	55 ± 16	0.20
Body mass index (kg/m^2^)	24.4 ± 3.1	24.7 ± 2.7	24.7 ± 3.8	23.2 ± 2.3	0.55
AF history (months)	24 (7–60)	24 (12–66)	24 (9–60)	36 (8–105)	0.54
AF triggers					0.34
Sympathetic activity	76 (90.5%)	30 (100%)	23 (88.5%)	10 (90.9%)	
Parasympathetic activity	8 (9.5%)	0	3 (11.5%)	1 (9.0%)	
AF episodes ≥ 1/week	45 (53.6%)	25 (83.3%)	14 (53.9%)	3 (27.3%)	0.005
AF at baseline lab. tests	11 (13.1%)	13 (43.3%)	7 (22.6%)	0	0.001
AF at ablation	12 (14.3%)	9 (30.0%)	4 (15.4%)	0	0.092
Atrial flutter	11 (13.1%)	4 (13.3%)	3 (11.5%)	0	0.65
NYHA class II	16 (19.0%)	11 (36.7%)	10 (38.5%)	0	0.018
Intense exercise (≥5 h/week)	5 (6.0%)	0	2 (7.7%)	2 (18.2%)	0.17
Hypertension	48 (57.1%)	14 (46.7%)	14 (53.8%)	4 (36.4%)	0.51
Dyslipidemia	28 (33.3%)	14 (46.7%)	8 (30.8%)	3 (27.3%)	0.50
Diabetes mellitus	9 (10.7%)	8 (26.7%)	2 (7.7%)	0	0.049
Hypoglycemic agents					
DPP 4 inhibitor	8 (9.5%)	7 (23.3%)	2 (7.7%)	0	
Biguanide	3 (3.5%)	2 (6.7%)	0	0	
Sulphonylurea	1 (1.2%)	1 (3.3%)	0	0	
Pioglitazone	1 (1.2%)	1 (3.3%)	0	0	
α-glucosidase inhibitor	0	3 (10.0%)	0	0	
Hemoglobin A1c (%)	6.0 ± 0.7	6.4 ± 1.7	6.1 ± 0.6	5.7 ± 0.5	0.37
Hemoglobin A1c in patients with diabetes mellitus (%)	7.0 ± 1.0	7.5 ± 2.4	6.9 ± 0.2	NA	0.82
SSS (HR at rest < 60 bpm or sinus pauses of > 3.0 s)	13 (15.5%)	10 (33.3%)	6 (23.1%)	2 (18.2%)	0.22
CHADS2 score	1.0 ± 1.0	1.2 ± 1.0	1.1 ± 1.0	0.5 ± 0.7	0.20
COPD	0	0	3 (11.5%)	0	0.002
Former smoker	53 (63.1%)	20 (66.7%	14 (53.8%)	4 (36.4%)	0.28
Current smoker	10 (11.9%)	2 (6.7%)	1 (3.8%)	0	0.38
History of PCI	1 (1.2%)	2 (6.7%)	0	0	0.23
Sleep apnea syndrome	5 (6.0%)	1 (3.3%)	1 (3.8%)	0	0.80
Cancer	6 (7.1%)	1 (3.3%)	2 (7.7%)	1 (9.1%)	0.87
Collagen disease	1 (1.2%)	0	0	0	0.85
C-reactive protein (mg/dL)	0.12 ± 0.21	0.09 ± 0.12	0.11 ± 0.09	0.06 ± 0.02	0.70
eGFR (mL/min/1.73 m^2^)	71 ± 14	71 ± 16	63 ± 15	74 ± 16	0.072
LVDd (mm)	47.9 ± 6.2	48.8 ± 5.0	50.5 ± 6.8	48.9 ± 4.6	0.30
Ejection fraction (%)	68 ± 6	65 ± 8	65 ± 10	67 ± 9	0.39
E/e’	7.5 ± 4.2	9.1 ± 4.8	8.8 ± 3.3	7.9 ± 3.8	0.30
LAVi ≥ 34.0 mm/m^2^	21 (26.6%)	11 (40.7%)	11 (42.3%)	2 (22.2%)	0.30
TRPG (mmHg)	18.0 ± 6.0	19.7 ±6.1	24.6 ± 8.3	18.0 ± 7.4	0.0006
Anti-arrhythmic drugs					
Amiodarone	11 (13%)	5 (17%)	6 (23%)	3 (27%)	0.49
Bepridil	3 (4%)	1 (3%)	1 (4%)	0	0.94
Sotalol	0	1 (3%)	1 (4%)	0	0.33
Cibenzoline	4 (5%)	2 (7%)	2 (8%)	0	0.78
Disopyramide	9 (11%)	2 (7%)	1 (4%)	1 (9%)	0.71
Flecainide	9 (11%)	6 (20%)	3 (12%)	2 (18%)	0.58
Pilsicainide	16 (19%)	8 (27%)	5 (19%)	1 (9%)	0.63
Propafenone	8 (10%)	4 (13%)	3 (12%)	1 (9%)	0.94
Aprindine	3 (4%)	0	1 (4%)	0	0.67
Other drugs					
Beta blocker	49 (58.3%)	15 (50.0%)	13 (50.0%)	7 (63.6%)	0.74
ACEI	6 (7.1%)	1 (3.3%)	3 (11.5%)	1 (9.1%)	0.69
ARB	27 (32.1%)	10 (33.3%)	9 (34.6%)	3 (27.2%)	0.98

ACEI, angiotensin converting enzyme inhibitor; AF, atrial fibrillation; ARB, angiotensin II receptor blocker; COPD, chronic obstructive pulmonary disease; DPP-4, dipeptidyl-peptidase 4; eGFR, estimated glomerular filtration rate; h, hour; HR, heart rate; lab., Laboratory; LAVi, left atrial volume indexed; LVDd, left ventricular end-diastolic diameter; PCI, percutaneous coronary intervention; SSS, sick sinus syndrome; TRPG, tricuspid regurgitation pressure gradient.

**Table 2:** BNP, ANP, and LAVi values in each group during follow-up.

	Baseline				1-Year				2-Year				3-Year			
	*Non-Recur*	*Short-Term*	*Mid-Term*	*Long-Term*	*Non-Recur*	*Short-Term*	*Mid-Term*	*Long-Term*	*Non-Recur*	*Short-Term*	*Mid-Term*	*Long-Term*	*Non-Recur*	*Short-Term*	*Mid-Term*	*Long-Term*
**BNP** (pg/mL)	26 (15–42)	46 (14–132)	40 (19–83)	23 (9–47)	19 (11–30)	NA	28 (17–55)	19 (9–28)	17 (9–30)	NA	22 (17–39)	8 (6–35)	16 (9–27)	NA	NA	13 (10–31)
**ANP** (pg/mL)	36 (22–61)	49 (27–106)	31 (22–64)	26 (15–43)	28 (18–38)	NA	39 (21–64)	30 (22–37)	24 (16–37)	NA	36 (27–53)	25 (18–40)	22 (16–38)	NA	NA	27 (18–43)
**LAVi** (mL/m^2^)	29.6 ± 10.7	36.2 ± 12.6	35.5 ± 14.3	28.5 ± 9.2	27.9 ± 8.9	NA	31.2 ± 9.4	25.8 ± 9.8	27.7 ± 9.0	NA	30.8 ± 7.9	28.4 ± 8.4	28.0 ± 8.1	NA	NA	28.5 ± 9.4

ANP, atrial natriuretic peptide; BNP, brain natriuretic peptide; LAVi, left atrial volume indexed; NA, not available.
